# Supplementary material for: The Archaeal Proteome Project advances knowledge about archaeal cell biology through comprehensive proteomics
Source: Nat Commun. 2020 Jun 19;11:3145. doi: 10.1038/s41467-020-16784-7 (PMC7305310; doi:10.1038/s41467-020-16784-7)
Supplement: Supplementary file 1 — Supplementary Information [file 41467_2020_16784_MOESM1_ESM.pdf]

The Archaeal Proteome Project advances knowledge about archaeal cell biology through comprehensive proteomics

Schulze et al. 2020

Supplementary information

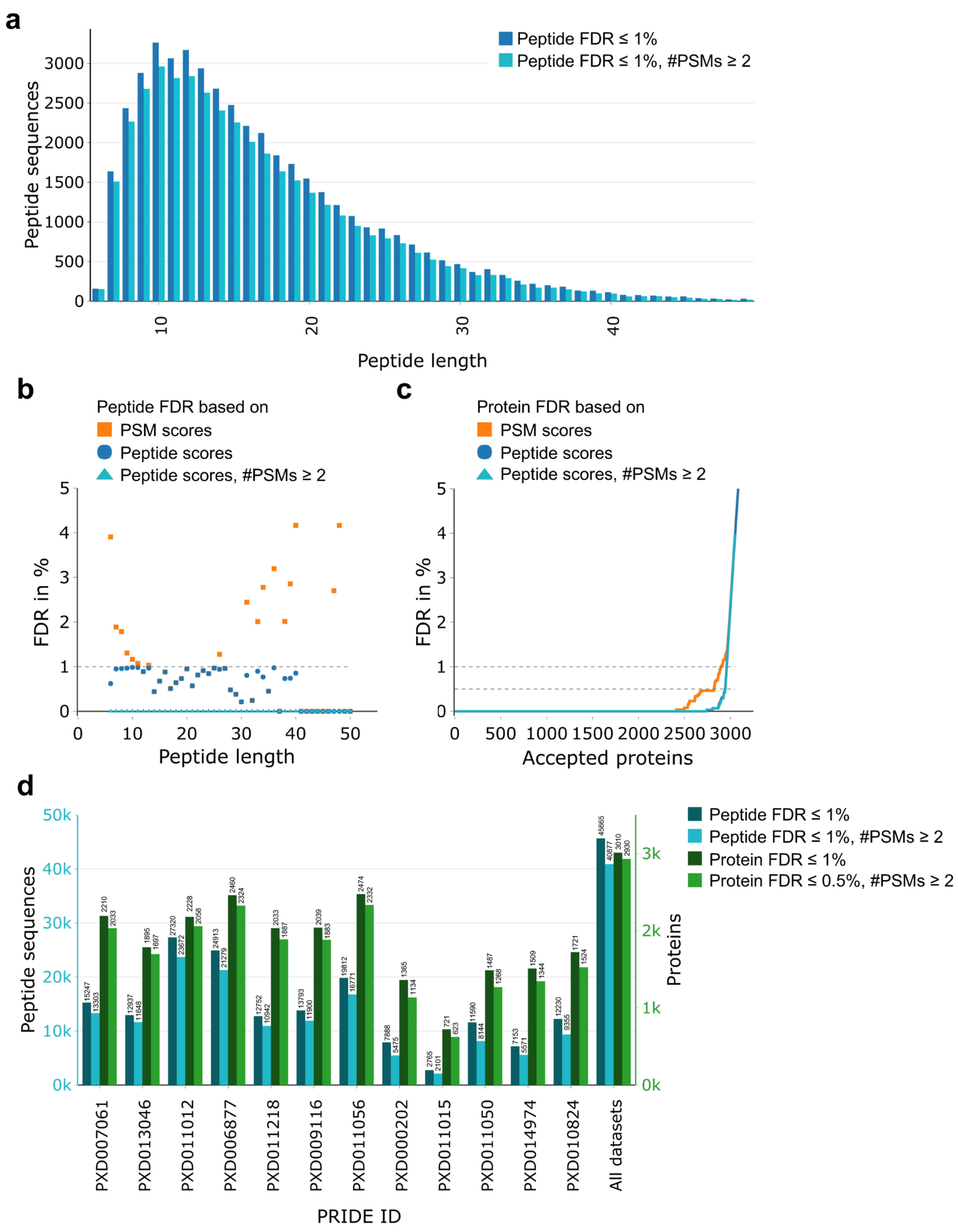

Supplementary Fig. 1 | Applying a threshold of two PSMs for each identification increases the confidence while only slightly decreasing the number of identifications. **a**, length distribution for peptide sequences identified based on a peptide FDR<=1% and applying (light blue) or omitting (dark blue) a threshold of two PSMs. **b**, For each peptide length, the FDR for all peptide sequences within this group was determined after (i) including all PSMs with a PEP≤1% (orange), (ii) adjusting the FDR on peptide level (dark blue) and (iii) filtering by a threshold of two PSMs (light blue). **c**, Protein FDRs are shown for the number of accepted proteins (ranked by protein q-value) after (i) including all PSMs with a PEP≤1% (orange), (ii) adjusting the FDR on protein level using the picked protein FDR approach (dark blue) and (iii) applying a threshold of two PSMs (light blue). **d**, The number of identified peptide sequences (blue) and proteins (green), with thresholds as indicated by the legend, is given for each dataset as well as the combination of all datasets. Source data are provided as a Source Data file.

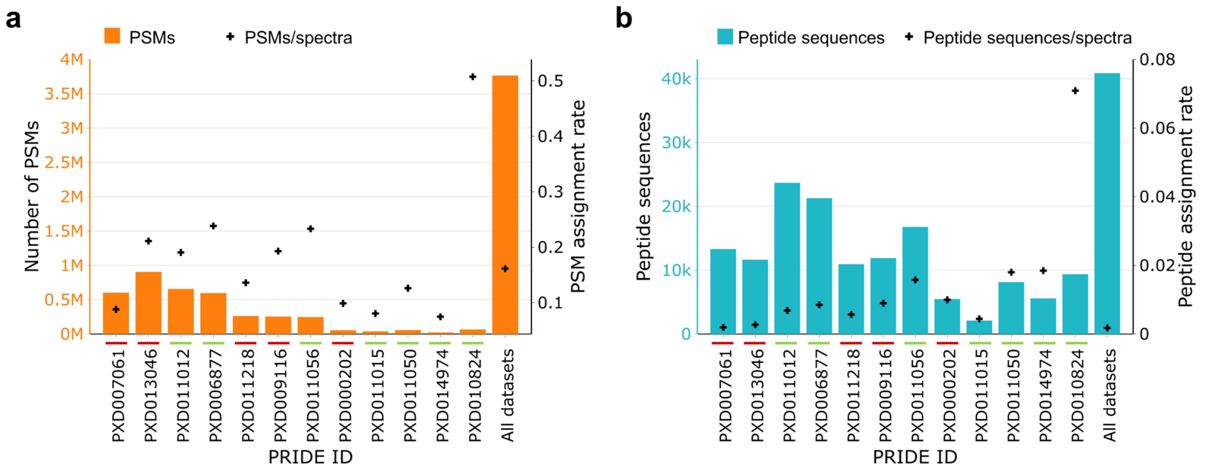

Supplementary Fig. 2 | High mass accuracy and sensitivity of the mass spectrometer leads to a higher identification rate. a, Number of PSMs (orange bars) and PSM identification rates (black cross) for each analyzed dataset. **b**, Number of peptide sequences (blue bars) and peptide identification rates (black cross) for each analyzed dataset. The type of mass spectrometer is indicated by red (LTQ Orbitrap series) and green (Q Exactive series and TripleTOF) lines, corresponding to comparatively low and high mass accuracy and sensitivity instruments, respectively. Source data are provided as a Source Data file.

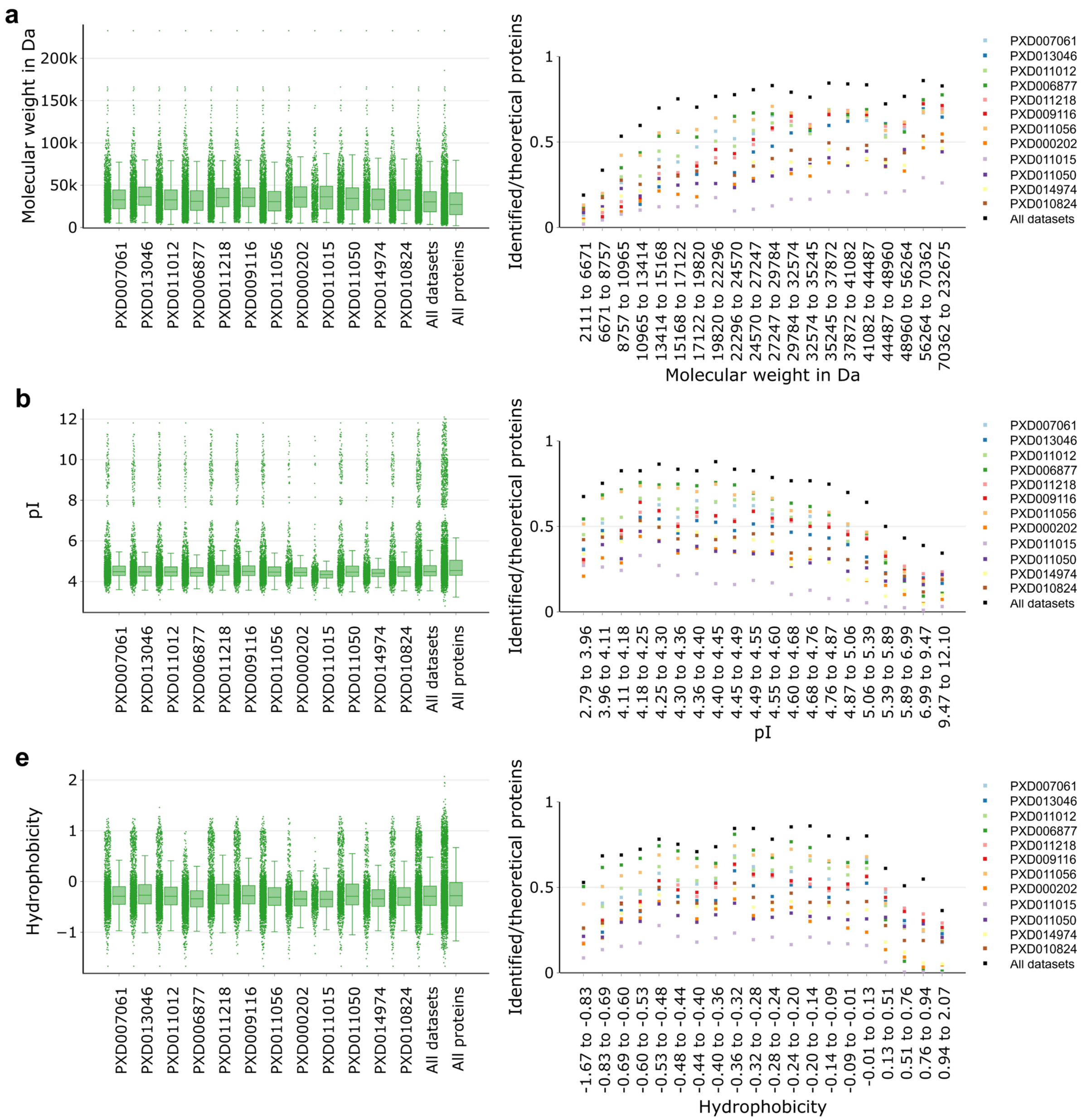

Supplementary Fig. 3 | The distribution of protein properties indicates biases against identification of low molecular weight, high pI and high hydrophobicity proteins. For each dataset, the combination of all datasets as well as the whole *H. volcanii* proteome, box-plots (left) of identified proteins (or theoretical proteins in the case of all proteins) are given for the molecular weight (**a**), pI (**b**) and hydrophobicity (**c**) (center line, median; box limits, upper and lower quartiles; whiskers, 1.5x interquartile range; points, all individual values). The number of identified proteins per dataset is given in Supplementary Fig. 1d, the total number of proteins in the theoretical proteome is 4074. Furthermore, for each property, the proteome was divided into 20 bins with an equal number of proteins (ranges for each bin indicated on the X axis) and for each bin the identification rate is given in a scatter plot (right). Source data are provided as a Source Data file.

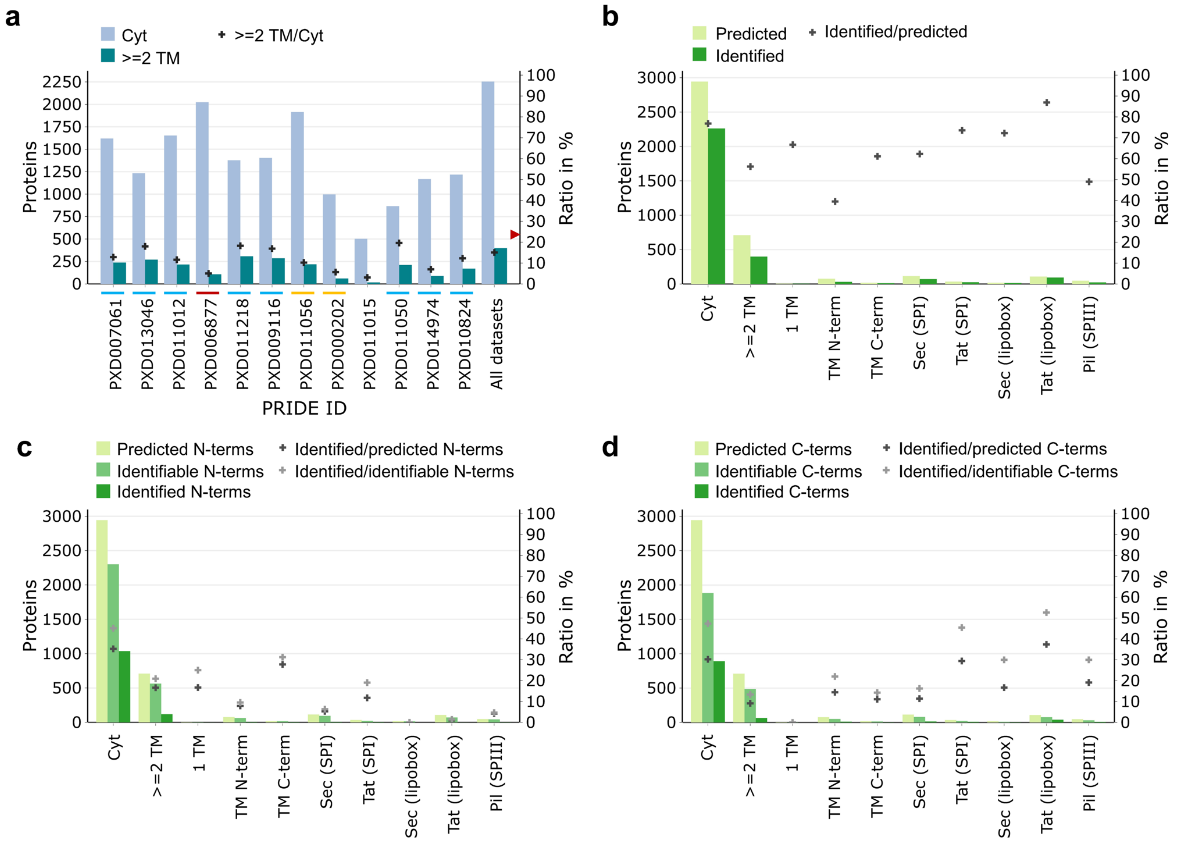

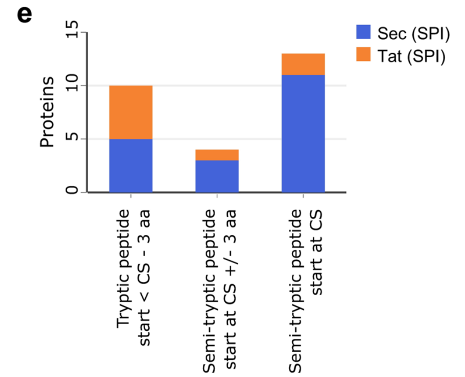

f

Secretory pathway	Previously described: Archaea	Previously described: *H. volcanii*	ArcPP: *H. volcanii*	Combined total
Sec (SPI)	12 (21)	1 (1)	14	25 (33)
Tat (SPI)	3 (7)	2 (4)	3	5 (8)

Supplementary Fig. 4 | Integral membrane proteins show a lower identification rate but a similar N-terminal maturation as cytosolic proteins. a, For each dataset, as well as the combination of all datasets, the number for identified cytosolic (light cyan) and integral membrane (dark cyan) proteins is shown as well as their ratio. The ratio of predicted integral membrane and cytosolic proteins for the whole proteome is 23% (red arrowhead). Datasets that used SDS, TRIzol and other detergents for sample preparation are marked with blue, red and orange lines, respectively. Further details about sample extraction and digestion are summarized in Supplementary Table 3. **b**, For each of the given categories (see Methods for more details), the number of predicted (light green) and identified (dark green) proteins is given, as well as the identification rate (cross). Cyt, cytosolic; TM, transmembrane domain; Sec, Sec pathway; Tat, twin arginine translocation pathway; Pil, type IV pilin pathway; SPI, signal peptidase I; lipobox, conserved cleavage site motif for lipoproteins; SPIII, signal peptidase III. Similarly, for each category, the number of predicted (light green), identifiable (trypsin or GluC cleavage site resulting in a terminal peptide of 6 - 50 amino acids, green) and identified (dark green) N-terminal (**c**) and C-terminal (**d**) peptides is given. Identification rates are shown based on the number of predicted (dark grey) or

identifiable (light grey) termini. **e**, Peptides within the first 60 amino acids of a protein, identified by semi-tryptic database search, were compared with SPI processing sites predicted by SignalP 5.0[1] for either Sec (blue) or Tat (orange) substrates. Results were differentiated into (i), proteins for which semi-tryptic peptides starting at the predicted CS were identified, indicating correct predictions; (ii), proteins with semi-tryptic peptides starting three amino acids before or after the predicted CS, regarded as refined predictions; and (iii), proteins for which a fully tryptic peptide starting at least three amino acids before the predicted CS was identified, suggesting potential false positive predictions. For the last category, it should be noted that post-translational secretion cannot be excluded. Therefore, proteins, for which the predicted processed N-termini would be hard to identify, due to too long or too short theoretical peptides, or modification by a lipid anchor as predicted by TatLipo[2], were not taken into account (Supplementary Note 3). **f**, For few archaeal proteins experimental support for their assigned secretion pathway (numbers in parenthesis) or their SPI CS (numbers without parenthesis) has been previously described (column Archaea). For only a minority of those, experimental characterization was performed in *H. volcanii* (column *H. volcanii*). This experimental support data is based on UniProt entries and corresponding publications for proteins listed in the training dataset of SignalP 5.0. For comparison, the number of processing sites identified within the ArcPP is given and the combined total is calculated. Source data are provided as a Source Data file.

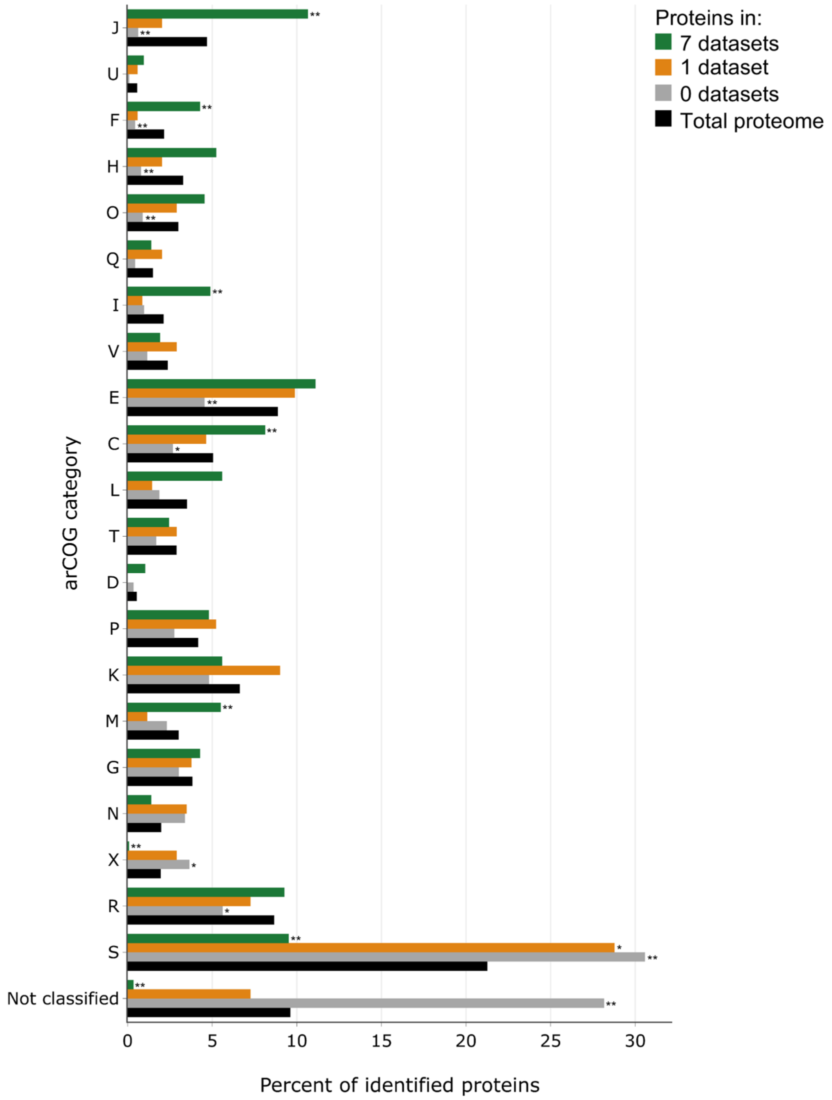

Supplementary Fig. 5 | arCOG categories representing crucial biological processes are enriched for proteins found in all whole cell proteomes, while poorly characterized proteins dominate the group of proteins found in only one dataset or not identified. Proteins identified in all whole cell proteome datasets (green, total = 1144), only one dataset (orange, total = 344) or not detected within the ArcPP (grey, total = 1122) were classified into corresponding arCOG categories (J, translation, ribosomal structure and biogenesis; U, intracellular trafficking, secretion, and vesicular transport; F, nucleotide transport and metabolism; H, coenzyme transport and metabolism; O, posttranslational modification, protein turnover, chaperones; Q, secondary metabolites biosynthesis, transport and catabolism; I, lipid transport and metabolism; V, defense mechanisms; E, amino acid transport and metabolism; C, energy production and conversion; L, replication, recombination and repair; T, signal transduction mechanisms; D, cell cycle control, cell division, chromosome partitioning; P, inorganic ion transport and metabolism; K, transcription; M, cell wall/membrane/envelope biogenesis; G, carbohydrate transport and metabolism; N, cell motility; X, mobilome; R, general function prediction only; S, function unknown; Not classified, no arCOG class determined). The distribution of arCOG categories for each group of proteins was compared to the distribution of arCOG categories within the whole *H. volcanii* proteome employing a two-sided Fisher's exact test on each arCOG category and applying Bonferroni correction for multiple testing. Significantly enriched or depleted arCOG categories are marked with asterisks (*, p-value < 0.05; **, p-value < 0.01). Source data are provided as a Source Data file.

a

Phylogenetic group	Number of homologs UreB	C	A	G	D	E	F
Archaea	203	716	219	198	201	131	201
Haloarchaea	112	106	104	108	106	80	106
Thaumarchaeota	68	535	93	69	71	48	75

PRIDE ID (growth media)	Number of PSMs UreB	C	A	G	D	E	F
PXD006877 (Hv-Min glycerol)	34	254	27	29	10	40	7
PXD013046 (Hv-Min lactic acid, succinic acid, glycerol)	-	143	-	2	57	-	-
PXD007061 (Hv-Min glucose)	-	2	-	-	-	-	-

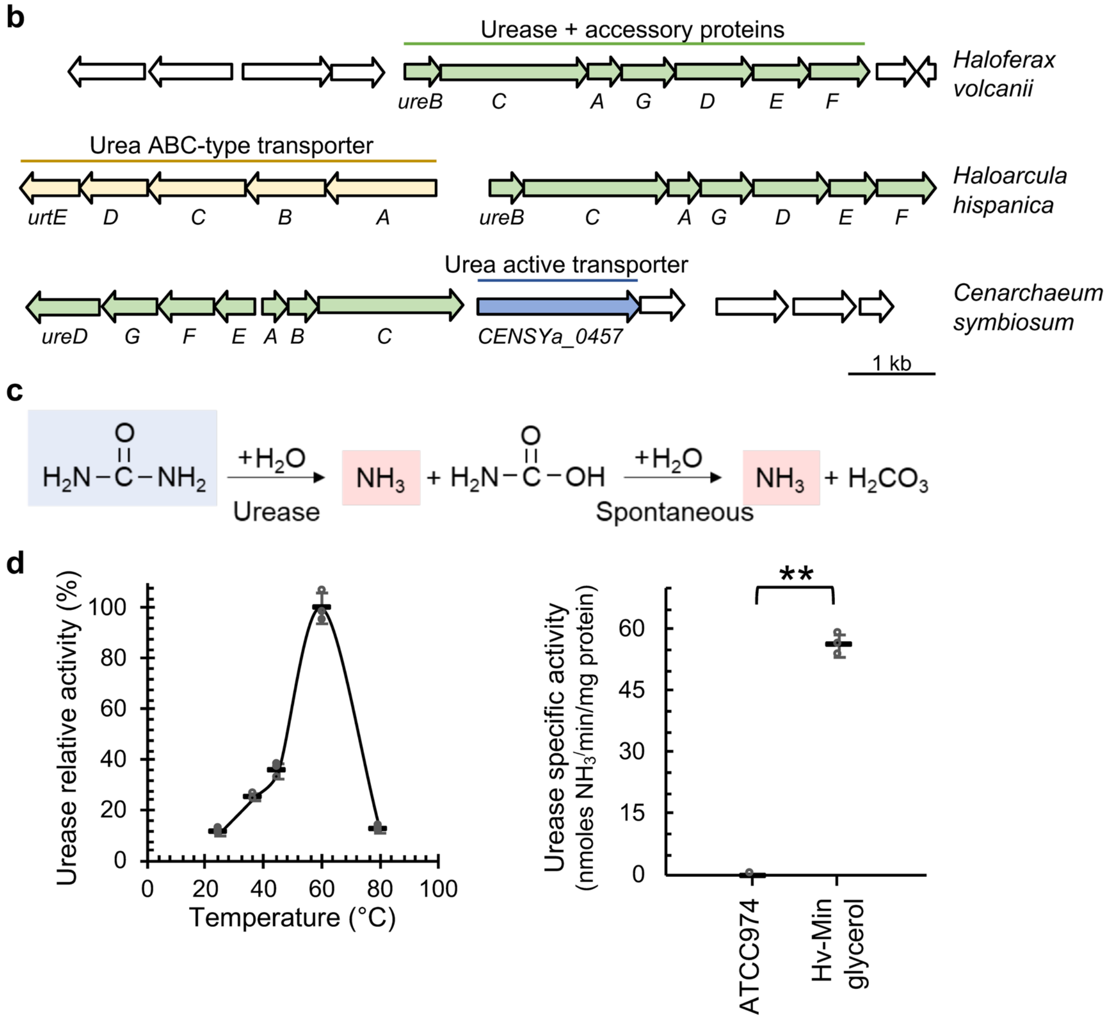

Supplementary Fig. 6 | Urease expression and activity in *H. volcanii* is dependent on growth conditions. a, The distribution of urease homologs in different archaeal phylogeny groups is given based on InterPro classification: UreB, urease beta subunit superfamily (IPR036461); UreC, urease alpha subunit family (IPR005848); UreA, urease gamma subunit superfamily (IPR036463); UreG, urease accessory protein UreG family (IPR004400); UreD, urease accessory protein UreD family (IPR002669); UreE, urease accessory protein UreE family (IPR012406) and UreF, UreF domain superfamily (IPR038277). Urease subunits have been identified within ArcPP; the number of peptide spectrum matches (PSMs) is given for each PRIDE dataset. It should be noted that PXD006877 is the only dataset that used glycerol minimal medium (GMM). **b**, The urease operon organization of *Haloferax volcanii* DS2

was compared to *Haloarcula hispanica* ATCC 33960 and the thaumarchaeote *Cenarchaeum symbiosum* A. **c**, Urease activity was detected by the phenol hypochlorite method with the substrate (urea, blue) and product (ammonia, red) indicated. **d**, Urease activity of *H. volcanii* was assayed for lysates of cells grown to log phase in GMM for different temperatures (left). Furthermore, urease activity was compared for lysates of cells grown to log-phase in GMM and complex medium (CM). The assay was performed at the 60 °C temperature optimum. The mean ± s.d. of all biological replicates is given (n=3), empty circles indicate individual measurements, and two asterisks indicate a p-value of 4×10^{-6} by a two-sided student t-test assuming equal variances. Source data are provided as a Source Data file.

Supplementary Table 1 | Archaeal proteomics datasets available on PRIDE.

Species	PRIDE ID
Candidatus Nanohaloarchaeum antarcticus	PXD010625
Halobacterium salinarum	PXD008466
Halobacterium salinarum	PXD003667
Haloferax mediterranei	PXD006211
Haloferax volcanii	PXD000202
Haloferax volcanii	PXD006877
Haloferax volcanii	PXD007061
Haloferax volcanii	PXD009116
Haloferax volcanii	PXD010824
Haloferax volcanii	PXD011012
Haloferax volcanii	PXD011015
Haloferax volcanii	PXD011050
Haloferax volcanii	PXD011056
Haloferax volcanii	PXD011218
Haloferax volcanii	PXD013046
Haloferax volcanii	PXD014974
Halohasta litchfieldiae	PXD010137
Halohasta litchfieldiae	PXD005076
Halorubrum lacusprofundi	PXD004202
Halorubrum lacusprofundi	PXD005092
Halorubrum lacusprofundi	PXD006515
Halorubrum lacusprofundi	PXD005076
Halorubrum lacusprofundi	PXD010625
Methanobacterium thermautotrophicus	PXD006685
Methanohalophilus portucalensis	PXD002024
Methanosarcina mazei	PXD004325
Methanothermobacter marburgensis	PXD003661
Nitrosopumilus maritimus	PXD007728
Nitrososphaera viennensis	PXD005297
Sulfolobus acidocaldarius	PXD009111
Sulfolobus acidocaldarius	PXD000289
Sulfolobus islandicus	PXD012246
Sulfolobus islandicus	PXD008644
Sulfolobus islandicus	PXD004179
Sulfolobus islandicus	PXD003424
Sulfolobus islandicus	PXD003074
Sulfolobus sulfactarius	PXD003282
Thermococcus gammatolerans	PXD000402
Various	PXD001860

For each species, the corresponding PRIDE IDs are listed. Notable datasets that are not deposited on PRIDE include [3–5].

Supplementary Table 2 | Strains analyzed in the ArcPP.

Strain	Background	Genotype	Plasmid	Reference
H26	DS70	DpHV2; DpyrE2		[6]
H119	DS70	DpHV2; DpyrE2; DtrpA; DleuB		[6]
HVLON3	H26	DpyrE2; PtnaA-lon-abi		[7]
HVABI	H26	DpyrE2; Dabi		[7]
MIG1	H26	DpyrE2; DrhoII		[8]
H53	DS70	DpHV2; DpyrE2; DtrpA		[6]
MT13	H53	DpHV2; DpyrE2; DtrpA	pTA963	[9]
aglB::trp	H53	DpHV2; DpyrE2; DtrpA; aglB::trp		[10]
AF103	H53	DpHV2; DpyrE2; DtrpA; DartA		[11]
FH54	H53	DpHV2; DpyrE2; DtrpA; Dhvo_1143		This work
FH26	H53	DpHV2; DpyrE2; DtrpA; Dhvo_0405	pFH25 (pTA963 with insert: p.tnaA::NdeI::hvo_0405 cds::GGP linker::6His::stop::EcoRI)	[12]
FH59	FH26	DpHV2; DpyrE2; DtrpA; Dhvo_0405	pJS151 (pTA963 with insert: p.tnaA::Hvo_0405 cds with coding bases 7-12 mutated from CGCCGC to AAGAAG (changes RR to LL)::GGP linker::6His tag::stop)	[12]
JS27	AF103	DpHV2; DpyrE2; DtrpA; DartA	pJS150 (pTA963 containing artAAlasmRS-GFP)	This work
RR01	AF103	DpHV2; DpyrE2 Dtrp; DartA	pRR01 (pTA963 containing artAC173AsmRS-GFP)	This work
RR02	AF103	DpHV2; DpyrE2 Dtrp; DartA	pRR02 (pTA963 containing artAR214A173AsmRS-GFP)	This work
RR03	AF103	DpHV2; DpyrE2 Dtrp; DartA	pRR03 (pTA963 containing artAR253AsmRS-GFP)	This work
LM08	H26	DpHV2; DpyrE2; DlysA; DargH		[13]
H26-pJAM1198	H26	DpHV2; DpyrE2	pJAM1198 (pJAM202c containing Flag-SAMP3 A90K)	[14]
HM1052-pJAM1198	H26	DpHV2; DpyrE2; DubaA	pJAM1198 (pJAM202c containing Flag-SAMP3 A90K)	[14]

Haloferax volcanii strains that were included in the ArcPP are given with their corresponding parental strains, genotype and, if applicable, plasmids that they have been transformed with.

Supplementary Note 1 | Comparison between original results and the reanalysis within the ArcPP. It should be noted that, while we tried to achieve a fair comparison between original search results and results from the reanalysis within the ArcPP (see Methods), limitations due to the use of different bioinformatic tools, including parameters that are not available for all search engines, the use of different protein databases as well as varying algorithms for statistical post-processing could not be evaded completely. In this light, a slight increase in PSMs for the overall dataset as well as increases by more than 10% for six datasets (Fig. 2a), is encouraging for a unified reanalysis, especially with a focus on confidence of identifications.

Additionally, even the decrease in identifications for three datasets can most likely be explained by the corresponding measurements and raw data processing. For PXD011015, since this dataset focused on identifying *N*-glycosylated peptides, in-source collision-induced dissociation was applied during most of the MS measurements and *N*-glycopeptides were selected for fragmentation by mass-tags[15]. However, the variable modifications corresponding to *N*-glycopeptides were not yet included in the reanalysis, leading to an altered target-decoy distribution and consequently lower identification rates even if PSMs with *N*-glycopeptide modifications from the original analysis were removed for the comparison. Conversely, differences for the datasets PXD013046 and PXD011218 are likely due to the fact that the original analysis with Proteome Discoverer included a spectral filtering step to remove noise peaks[16,17]. This step was omitted in the reanalysis. The combination of lower mass accuracy of the measurement and the stronger focus on a stringent control of the PEP in the reanalysis might have led to the lower number of observed PSMs and peptide identifications.

Supplementary Note 2 | Insights into the optimization of sample processing. While it should be noted that the comparison of sample processing and experimental setups is a multivariate problem, some general conclusions could be drawn from this large-scale analysis. As expected, high-resolution mass spectrometers (QExactive series and TripleTOF) achieve higher identification rates than ion trap instruments (LTQ Orbitrap series), which have comparatively lower resolution and sensitivity (Supplementary Fig. 2). Longer HPLC-gradients (more than 2h) on LTQ Orbitrap series instruments led to a higher number of PSMs but did not provide any advantage for the peptide identification rate, indicating that the sensitivity rather than instrument cycle time was the major limitation. The role of cellular fractionation is hard to evaluate in this context, but peptide fractionation by SCX chromatography (PXD006877) or high-pH reversed-phase fractionation (PXD011056) resulted in the highest number of protein identifications. SCX chromatography, however, had an advantage over high-pH reversed-phase fractionation in the number of peptide identifications, as would be expected since SCX is the more orthogonal chromatography of the two in combination with the C18 reversed-phase chromatography that is coupled to the MS. Interestingly though, the use of multiple proteases (trypsin and GluC) yielded more peptide identifications (and thus a higher sequence coverage) than any other experimental setup, even without fractionation (PXD011012).

When analyzing different protein characteristics of identified and missing proteins, further potential biases could be identified. The molecular weight distribution of identified proteins showed a strong decrease in identification rates for proteins <13 kDa (Supplementary Fig. 3a), revealing problems in the identification of small proteins to a similar degree for all datasets. On

the other hand, in regard to the isoelectric point (pI) and hydrophobicity, proteins with a pI >5 (Supplementary Fig. 3b) or a hydrophobicity >0 (Supplementary Fig. 3c) showed a reduced identification rate. In both cases, this effect was most pronounced in the dataset PXD006877, which used protein extraction by TRIzol and otherwise showed the highest number of protein identifications (together with PXD011056). Since these results correspond to known challenges in the identification of integral membrane proteins, we compared identifications for proteins with at least two predicted TM domains (as well as other categories, see Methods). Indeed, samples prepared by TRIzol extraction showed the lowest identification rate for integral membrane proteins. In contrast, samples solubilized by SDS and further processed by SDS-PAGE showed the highest numbers of identified membrane proteins (Supplementary Fig. 4a). However, it should be noted that these were also datasets that performed cell fractionation and analyzed membrane fractions. Furthermore, these comparisons do not account for negative combinations of protein properties. For example, the very low identification rate for proteins with a single N-terminal TM domain (Supplementary Fig. 4b) can be attributed to the fact that about half of these are very short proteins.

Supplementary Note 3 | Semi-enzymatic protein database search confirms, refines and contradicts different predicted signal peptidase cleavage sites. The majority of cell surface proteins contains N-terminal signal peptides, which are processed by SPI, SPII (lipobox) or SPIII (prepilin peptidase) upon transport through the Sec or Tat pathway. Results of a semi-enzymatic database searches were compared to signal peptide cleavage sites (CS) predicted by SignalP 5.0[1]. SignalP 5.0 is the only engine allowing for the prediction of Sec (SPI) and Tat (SPI) substrates for archaea, however, it is not trained on Tat substrates containing a lipobox. Therefore, results from TatLipo[2] were taken into account, overriding Tat (SPI) substrates as Tat (lipobox).

In total, 13 SPI processing sites, corresponding to 11 Sec and two Tat substrates, were confirmed by semi-tryptic peptides (Supplementary Fig. 4e). Additionally, four SPI processing sites, corresponding to three Sec and one Tat substrate(s), were within a range of +/- three amino acids and could be regarded as refined CS. This represents a six-fold increase over previously experimentally verified CS in *H. volcanii*, and a two-fold increase for archaea overall (Supplementary Fig. 4f), illustrating the strength of a combined proteomic analysis even without dedicated methods for N-terminal peptide identification like TAILS[18]. Furthermore, fully enzymatic peptides starting at least five amino acids N-terminal of the predicted CS and lacking semi-tryptic peptides after signal peptide cleavage were identified for five Sec and 20 Tat substrates. Two of those Tat substrates contain a lipobox, and lipidation of the mature protein would prohibit the semi-tryptic N-terminal peptide to be identified (exact mass of the PTM yet unknown; also, the lipid-modified peptides may be so hydrophobic that they are not efficiently eluted from C18 reverse phase columns). Similarly, for 13 proteins, all of which are predicted to be Tat substrates, the new theoretical N-terminus would consist of tryptic peptides that would be too short or too long for identification. Therefore, it is conceivable that the identified fully-tryptic peptides represent the N-termini of proteins before their post-translational transport and processing.

Interestingly, for two Tat and two Sec substrates predicted to be processed by SPI, N-terminal peptides of the pre-proteins as well as semi-tryptic peptides of the predicted mature protein were identified. These data are consistent with post-translational translocation of these proteins with stable, proteomically identifiable intermediates. While H. volcanii Tat-precursor proteins have previously been identified[12], much less is known about post-translational transport of Sec substrates[19]. Our proteomics data might thus represent the first report of post-translationally transported archaeal proteins. Alternatively, it is possible that processing rates for co-translationally transported Sec (SPI) substrates in *H. volcanii* are slower than in bacteria[20], allowing for the identification of N-termini of proteins that are not fully transcribed yet. However, for five Sec and five Tat substrates only fully-enzymatic peptides N-terminal of the predicted CS and no semi-tryptic peptides have been identified, despite no apparent reason for why the predicted processed N-termini should not be identifiable (Supplementary Fig. 4e). This indicates that these proteins are likely incorrectly predicted to be secreted through either pathway. Altogether, these results will allow for the optimization of archaeal signal peptide prediction programs and provide valuable insights into archaeal cell surface biogenesis.

Supplementary Note 4 | Identification of probable RhoII and LonB substrates through the reanalysis of datasets over/under-expressing membrane proteases. In the dataset PXD011218, the proteome of a wt strain was compared with a deletion strain of *rhoII*, identifying 37 potential RhoII targets[17]. The reanalysis within the ArcPP resulted in the identification of five additional proteins that were present in at least two replicates of the mutant but not the wt strain. These included four integral membrane proteins: two different ABC transport systems (HVO_A0147 and A0338), a major facilitator family transport protein (HVO_2578) and a hypothetical protein (HVO_A0497). The amino acid sequences of the four integral membrane proteins were analyzed and all of them evidenced the rhomboid protease recognition motif[21], suggesting that these proteins indeed represent RhoII substrates, which had escaped identification in the preceding studies.

Similarly, we reanalysed the datasets comparing proteomes of *H. volcanii* cells containing reduced versus physiological LonB content (PXD013046 and PXD007061)[16,22]. In PXD007061, two (HVO_2447A, HVO_2517) and one (HVO_A0574) of the previously unidentified proteins were predominantly detected in at least two replicates of cells with reduced LonB expression and physiological protease concentrations, respectively, suggesting a link to LonB regulation.

Notably, while for these novel potential membrane protease targets no connection was found to the known phenotypes displayed of the RhoII or LonB mutant strains, most of these candidates are proteins of unknown function, thereby providing hints towards an improved functional annotation of archaeal proteomes.

Almost all the candidate target proteins that were identified in our previous studies were also detected in the reanalysis, except two for RhoII (HVO_1210 (flgA1) and HVO_1153 (hypothetical)) and one for LonB (HVO_A0418 (hypothetical)).

Supplementary Note 5 | Most proteins which have not yet been detected have properties complicating their identification. A total of 1122 proteins from the theoretical proteome have not been identified in any dataset. A large fraction of those (770, representing 69% of the non-identified proteins) have physicochemical parameters which are associated with a reduced identification rate (<13 kDa, pI >5.5; GRAVY >0 or combinations thereof). Improving the sample preparation and MS measurements accordingly would therefore likely allow for their detection.

In addition, we detected four islands with a low identification rate (below 40%; this explains 135 out of 1122 non-identified proteins). Plasmid pHV1 is the first island as it has a low identification rate of 37.5% (36 identified proteins of 96 genes), which is especially low near HVO_C0008 to HVO_C0018. Two other islands correspond to predicted proviruses (HVO_A0216 to HVO_A0256; HVO_A0005 to HVO_A0062). The fourth island (HVO_B0160 - HVO_B0181) is adjacent to the genes coding for respiratory nitrate reductase (HVO_B0161 - HVO_B0166), which also has not been identified. This region had been analyzed previously[23] and nitrate reductase transcription and activity was only detected under anaerobic conditions, for which a proteomic analysis is missing so far. The transcription regulator NarO is constitutively expressed[23] and has been identified in two whole proteome datasets.

Among the residual 298 non-identified proteins are 52 pseudogenes, 11 transposases, and 24 proteins which are assigned to four additional provirus candidates. Of the remainder (209), only 119 are encoded on the chromosome, while 50 are encoded on pHV4 and 40 on pHV3. Interestingly, eight non-identified proteins in the genomic region HVO_1205 to HVO_1221 are related to motility and chemotaxis. While it had been shown that motility in *H. volcanii* depends on media and growth conditions[24], this process and contributing factors are not fully understood yet.

Supplementary Note 6 | Identified proteins encoded by genes which are disrupted. In our annotation, all products of disrupted genes are tagged by the term nonfunctional, which allowed their identification in the lists with identified proteins. Commonly, it would be expected that stable proteins, detectable by proteomics, are not generated from disrupted genes. Thus, we have analyzed these cases in detail.

(i) HVO_0712, aroE, shikimate dehydrogenase: This gene has two frameshifts in the original genome sequence (CP001956)[25] as revealed by comparison to resequencing results for the same strain (AOHU01000097)[26]. The non-disrupted version is C498_15168 (UniProt: L9UNH6). Thus, biologically, HVO_0712 is a functional protein, consistent with its identification in 5 whole proteome datasets.

(ii) HVO_A0006, HVO_1151, HVO_1838 and HVO_1911: These four genes are disrupted either by frameshift or by transposon targeting. These disruptions are considered biological as an identical disruption was detected upon resequencing of this strain. In all four cases, all the identified peptides were upstream of the frameshift/targeting position. As gene disruption by frameshift or transposon targeting leaves all transcription and translation signals intact, it is possible that the gene is transcribed into mRNA, followed by translation, but with an aberrant C-terminal extension up to the next in-frame stop codon. If the resulting translation product is

stable enough, it can be detected by proteomics. These proteins were detected in one (HVO_A0006, HVO_1151), two (HVO_1838) or three (HVO_1911) datasets. It should be noted that HVO_A0006 had been implicated in DNA A-methylation[27] but in a follow-up study, this was rated to have been a false positive result caused by an insufficient sequencing coverage[28].

(iii) HVO_2176: The coding region starts very close to the N-terminus of the functional ortholog HFX_2235 (UniProt: I3R6R3) from *H. mediterranei*. Two peptides have been identified for this protein. However, there is no potential start codon (ATG, GTG, TTG) between the first identified peptide and the next upstream in-frame stop codon. As the genome region is identical to the resequenced version, a genome sequence error is very unlikely. The most likely possibility is that another codon (e.g. ATA) functions as a start codon in this case. Unfavorably placed Lys/Arg and Glu residues preclude the identification of the N-terminal peptide in a trypsin or GluC digest.

(iv) HVO_B0311, HVO_2444, HVO_2090A and HVO_A0370: As described in the methods, FDR calculations were dataset-specific, which leads to a small number of proteins being identified only in the combined dataset but not in any individual dataset and vice versa. Such cases may represent false positive identifications or may indicate very low protein amounts. The nonfunctional proteins HVO_B0311 and HVO_2444 were identified in a single dataset but were not significant in the combined dataset. HVO_2090A was identified in 3 datasets but was not significant in the combined dataset. HVO_A0370 was significant in the combined dataset but in none of the individual datasets.

(v) HVO_D0001: This gene is encoded in plasmid pHV2, but its classification as nonfunctional is a technical artefact (caused by opening of the plasmid ring within this gene, which disconnects the two parts of the coding region). Plasmid pHV2 is present in the wildtype strain DS2 but has been cured away while developing laboratory strains. Thus, the plasmid is absent from all strains that have been subjected to proteomic analysis. However, the replication origin of pHV2, which covers most of HVO_D0001, is the basis for many plasmids used for complementation and homologous protein expression. This includes pTA963 (GenBank: FN645890)[29] which was present in the proteomically analyzed strains. There are sequence differences between FN645890 and pHV2 (CP001954)[25].

Supplementary Note 7 | Overview of the genetic lineage of *Haloferax volcanii* strains included in the ArcPP. We briefly summarize the various strains which were used for the proteome datasets. All are based on the wildtype isolate of *Haloferax volcanii* (strain DS2) (ATCC 29605, NCIMB 2012)[30], which was used for genomic sequencing[25] and is therefore the basis for the theoretical proteome. Strain DS2 was cured of plasmid pHV2 without mutagenesis, resulting in DS70[31]. Three genes were deleted from DS70 as selection markers[6], resulting successively in strain H26 (Δ*pyrE2*), H53 (Δ*pyrE2*, Δ*trpA*) and H119 (Δ*pyrE2*, Δ*trpA*, Δ*leuB*). These were the parent strains for further deletion/expression of genes under study (see the corresponding references for details of strain generation and Supplementary Table 2): HVLON3 (PtnaA-*lon*-*abi*) and HVABI (Δ*abi*) from H26; MIG1 (Δ*rhoII*) from H26; Δ*ubaA*+FlagSAMP3 from H26, *aglB*::*trpA* from H53; MT13, AF103, FH26, FH54, FH59, RR01-3, JS27 from H53. Independently, strain H26 was converted to LM08 by deletion of *lysA* and *argH*[13].

Supplementary References

1. Almagro Armenteros, J. J. *et al.* SignalP 5.0 improves signal peptide predictions using deep neural networks, *Nat Biotechnol* **37,** 420–423 (2019).
2. Storf, S. *et al.* Mutational and Bioinformatic Analysis of Haloarchaeal Lipobox-Containing Proteins, *Archaea* **2010,** 11 (2010).
3. Bjellqvist, B. Basse, B. Olsen, E. & Celis, J. E. Reference points for comparisons of two-dimensional maps of proteins from different human cell types defined in a pH scale where isoelectric points correlate with polypeptide compositions, *Electrophoresis* **15,** 529–539 (1994).
4. Soto, D. F. *et al.* Global effect of the lack of inorganic polyphosphate in the extremophilic archaeon Sulfolobus solfataricus: A proteomic approach, *J Proteomics* **191,** 143–152 (2019).
5. Feng, J. *et al.* Proteomic analysis of the secretome of haloarchaeon Natrinema sp. J7-2, *J Proteome Res* **13,** 1248–1258 (2014).
6. Allers, T. Ngo, H.-P. Mevarech, M. & Lloyd, R. G. Development of additional selectable markers for the halophilic archaeon Haloferax volcanii based on the leuB and trpA genes, *Appl Environ Microbiol* **70,** 943–953 (2004).
7. Cerletti, M. *et al.* The LonB protease controls membrane lipids composition and is essential for viability in the extremophilic haloarchaeon Haloferax volcanii, *Environ Microbiol* **16,** 1779–1792 (2014).
8. Parente, J. *et al.* A rhomboid protease gene deletion affects a novel oligosaccharide N-linked to the S-layer glycoprotein of Haloferax volcanii, *J Biol Chem* **289,** 11304–11317 (2014).
9. Tripepi, M. Esquivel, R. N. Wirth, R. & Pohlschröder, M. Haloferax volcanii cells lacking the flagellin FlgA2 are hypermotile, *Microbiology* **159,** 2249–2258 (2013).
10. Abu-Qarn, M. & Eichler, J. Protein N-glycosylation in Archaea: defining Haloferax volcanii genes involved in S-layer glycoprotein glycosylation, *Mol Microbiol* **61,** 511–525 (2006).
11. Abdul Halim, M. F. *et al.* Haloferax volcanii archaeosortase is required for motility, mating, and C-terminal processing of the S-layer glycoprotein, *Mol Microbiol* **88,** 1164–1175 (2013).
12. Abdul Halim, M. F. *et al.* ArtA-Dependent Processing of a Tat Substrate Containing a Conserved Tripartite Structure That Is Not Localized at the C Terminus, *J. Bacteriol.* **199,** e00802-16 (2017).
13. McMillan, L. J. *et al.* Multiplex quantitative SILAC for analysis of archaeal proteomes: a case study of oxidative stress responses, *Environ Microbiol* **20,** 385–401 (2018).
14. Miranda, H. V. *et al.* Archaeal ubiquitin-like SAMP3 is isopeptide-linked to proteins via a UbaA-dependent mechanism, *Mol Cell Proteomics* **13,** 220–239 (2014).
15. Esquivel, R. N. Schulze, S. Xu, R. Hippler, M. & Pohlschroder, M. Identification of Haloferax volcanii Pilin N-Glycans with Diverse Roles in Pilus Biosynthesis, Adhesion, and Microcolony Formation, *J Biol Chem* **291,** 10602–10614 (2016).
16. Cerletti, M. Paggi, R. A. Guevara, C. R. Poetsch, A. & Castro, R. E. de. Global role of the membrane protease LonB in Archaea: Potential protease targets revealed by quantitative proteome analysis of a lonB mutant in Haloferax volcanii, *J Proteomics* **121,** 1–14 (2015).
17. Costa, M. I. *et al.* Haloferax volcanii Proteome Response to Deletion of a Rhomboid Protease Gene, *J Proteome Res* **17,** 961–977 (2018).

18. Kleifeld, O. *et al.* Identifying and quantifying proteolytic events and the natural N terminome by terminal amine isotopic labeling of substrates, *Nat Protoc* **6,** 1578–1611 (2011).

19. Irihimovitch, V. & Eichler, J. Post-translational secretion of fusion proteins in the halophilic archaea Haloferax volcanii, *J Biol Chem* **278,** 12881–12887 (2003).

20. Pohlschröder, M. Murphy, C. & Beckwith, J. In Vivo Analyses of Interactions between SecE and SecY, Core Components of the Escherichia coli Protein Translocation Machinery, *Journal of Biological Chemistry* **271,** 19908–19914 (1996).

21. Strisovsky, K. Sharpe, H. J. & Freeman, M. Sequence-specific intramembrane proteolysis: identification of a recognition motif in rhomboid substrates, *Mol Cell* **36,** 1048–1059 (2009).

22. Cerletti, M. *et al.* LonB Protease Is a Novel Regulator of Carotenogenesis Controlling Degradation of Phytoene Synthase in Haloferax volcanii, *J Proteome Res* **17,** 1158–1171 (2018).

23. Hattori, T. *et al.* Anaerobic Growth of Haloarchaeon Haloferax volcanii by Denitrification Is Controlled by the Transcription Regulator NarO, *J Bacteriol* **198,** 1077–1086 (2016).

24. Tripepi, M. Imam, S. & Pohlschröder, M. Haloferax volcanii flagella are required for motility but are not involved in PibD-dependent surface adhesion, *J Bacteriol* **192,** 3093–3102 (2010).

25. Hartman, A. L. *et al.* The complete genome sequence of Haloferax volcanii DS2, a model archaeon, *PLoS One* **5,** e9605 (2010).

26. Becker, E. A. *et al.* Phylogenetically driven sequencing of extremely halophilic archaea reveals strategies for static and dynamic osmo-response, *PLoS Genet* **10,** e1004784 (2014).

27. Ouellette, M. Jackson, L. Chimileski, S. & Papke, R. T. Genome-wide DNA methylation analysis of Haloferax volcanii H26 and identification of DNA methyltransferase related PD-(D/E)XK nuclease family protein HVO_A0006, *Front Microbiol* **6,** 251 (2015).

28. Ouellette, M. Gogarten, J. P. Lajoie, J. Makkay, A. M. & Papke, R. T. Characterizing the DNA Methyltransferases of Haloferax volcanii via Bioinformatics, Gene Deletion, and SMRT Sequencing, *Genes (Basel)* **9** (2018).

29. Allers, T. Barak, S. Liddell, S. Wardell, K. & Mevarech, M. Improved strains and plasmid vectors for conditional overexpression of His-tagged proteins in Haloferax volcanii, *Appl Environ Microbiol* **76,** 1759–1769 (2010).

30. Mullakhanbhai, M. F. & Larsen, H. Halobacterium volcanii spec. nov. a Dead Sea halobacterium with a moderate salt requirement, *Arch Microbiol* **104,** 207–214 (1975).

31. Wendoloski, D. Ferrer, C. & Dyall-Smith, M. L. A new simvastatin (mevinolin)-resistance marker from Haloarcula hispanica and a new Haloferax volcanii strain cured of plasmid pHV2, *Microbiology* **147,** 959–964 (2001).
