# Supplementary material for: The Archaeal Proteome Project advances knowledge about archaeal cell biology through comprehensive proteomics
Source: Nat Commun. 2020 Jun 19;11:3145. doi: 10.1038/s41467-020-16784-7 (PMC7305310; doi:10.1038/s41467-020-16784-7)
Supplement: Supplementary file 3 — Description of Additional Supplementary Files [file 41467_2020_16784_MOESM3_ESM.docx]

**Description of Additional Supplementary Files**

**File Name: Supplementary Data 1**

**Description:** Experimental meta data for datasets within the ArcPP. For each dataset (given by PRIDE ID), the analyzed strains (see Supplementary Table 2 for details), growth conditions (growth media, supplements added to the media, growth temperature and growth phase at the time of taking the sample) and MS sample preparations (cellular fraction, cell lysis, labeling, alkylation of proteins, enzymes used for digestion, fractionation or enrichment of peptides or proteins and additional sample preparation steps) are listed.

**File Name: Supplementary Data 2**

**Description:** LC-MS/MS parameters for datasets included in the ArcPP. For each dataset (given by PRIDE ID), the HPLC column, gradient and separation temperature as well as important MS settings are given. The total number of spectra was calculated based on mzML files generated from raw result files.

**File Name: Supplementary Data 3**

**Description:** Datasets included in the ArcPP. Datasets analyzed within the ArcPP are listed with the corresponding laboratory (corresponding author), PRIDE ID, the year and month of dataset submission to PRIDE as well as an FTP URL and PubMed ID, if available.

**File Name: Supplementary Data 4**

**Description:** Plasmid sequences for the generation of overexpression strains. Sequences for the plasmids pJS150, pRR01, pRR02 and pRR03 that have been transformed into AF103 in order to generate the overexpression strains JS27, RR02, RR02 and RR03, respectively, are provided as gb files.
