# Supplementary material for: The Archaeal Proteome Project advances knowledge about archaeal cell biology through comprehensive proteomics
Source: Nat Commun. 2020 Jun 19;11:3145. doi: 10.1038/s41467-020-16784-7 (PMC7305310; doi:10.1038/s41467-020-16784-7)
Supplement: Supplementary file 8 — Reporting Summary [file 41467_2020_16784_MOESM8_ESM.pdf]

nature research

Corresponding author(s): Mechthild Pohlschroder

Last updated by author(s): May 10, 2020

Reporting Summary

Nature Research wishes to improve the reproducibility of the work that we publish. This form provides structure for consistency and transparency in reporting. For further information on Nature Research policies, see Authors & Referees and the Editorial Policy Checklist.

Statistics

For all statistical analyses, confirm that the following items are present in the figure legend, table legend, main text, or Methods section.

n/a	Confirmed	
☐	☒	The exact sample size (*n*) for each experimental group/condition, given as a discrete number and unit of measurement
☐	☒	A statement on whether measurements were taken from distinct samples or whether the same sample was measured repeatedly
☐	☒	The statistical test(s) used AND whether they are one- or two-sided<br>*Only common tests should be described solely by name; describe more complex techniques in the Methods section.*
☒	☐	A description of all covariates tested
☐	☒	A description of any assumptions or corrections, such as tests of normality and adjustment for multiple comparisons
☐	☒	A full description of the statistical parameters including central tendency (e.g. means) or other basic estimates (e.g. regression coefficient) AND variation (e.g. standard deviation) or associated estimates of uncertainty (e.g. confidence intervals)
☐	☒	For null hypothesis testing, the test statistic (e.g. *F*, *t*, *r*) with confidence intervals, effect sizes, degrees of freedom and *P* value noted<br>*Give P values as exact values whenever suitable.*
☐	☒	For Bayesian analysis, information on the choice of priors and Markov chain Monte Carlo settings
☐	☒	For hierarchical and complex designs, identification of the appropriate level for tests and full reporting of outcomes
☒	☐	Estimates of effect sizes (e.g. Cohen's *d*, Pearson's *r*), indicating how they were calculated

Our web collection on statistics for biologists contains articles on many of the points above.

Software and code

Policy information about availability of computer code

Data collection	Data was collected from the public repositories PRIDE and jPOST. No software has been used for data acquisition.
Data analysis	Python3.5/3.6/3.7, Ursgal v0.6.5/v0.6.6, X!Tandem version Vengeance, MS-GF+ version 2019.04.18, MSFragger version 20190222, ThermoRawFileParser v1.1.2, ProteoWizard/msConvert 3.0.19046, Percolator version 3.4.0, TMHMM 2.0, SignalP 5.0, FlaFind, TatFind, LipoP 1.0, TatLipo, plotly 3.10.0, Microsoft Excel 365, custom code uploaded to https://github.com/arcpp/ArcPP

For manuscripts utilizing custom algorithms or software that are central to the research but not yet described in published literature, software must be made available to editors/reviewers. We strongly encourage code deposition in a community repository (e.g. GitHub). See the Nature Research guidelines for submitting code & software for further information.

Data

Policy information about availability of data

All manuscripts must include a data availability statement. This statement should provide the following information, where applicable:

- Accession codes, unique identifiers, or web links for publicly available datasets
- A list of figures that have associated raw data
- A description of any restrictions on data availability

The raw files of all proteomic datasets are available on PRIDE with the following identifiers: PXD000202, PXD006877, PXD007061, PXD009116, PXD010824, PXD011012, PXD011015, PXD011050, PXD011056, PXD011218, PXD012683, PXD013046. The annotated proteome of H. volcanii is deposited at 10.5281/zenodo.3565580. PSMs and summarized result files for all datasets are deposited at 10.5281/zenodo.3724743. Furthermore, all main result files and all meta data is available at https://github.com/arcpp/ArcPP. The source data underlying all figures are provided as a Source Data file.

Field-specific reporting

Please select the one below that is the best fit for your research. If you are not sure, read the appropriate sections before making your selection.

☒ Life sciences ☐ Behavioural & social sciences ☐ Ecological, evolutionary & environmental sciences

For a reference copy of the document with all sections, see nature.com/documents/nr-reporting-summary-flat.pdf

Life sciences study design

All studies must disclose on these points even when the disclosure is negative.

Sample size	12 proteomic datasets for H. volcanii were chosen based on their availability and quality of MS measurements. These datasets comprise more than 23 Mio. spectra.
Data exclusions	Datasets measured on instruments older than the LTQ Orbitrap series (or corresponding instruments from other vendors) were not taken into account due to their low mass accuracy and sensitivity. Only measurements for H. volcanii were analyzed. Files based on data independent acquisition techniques were excluded from analysis. These criteria were established before the start of the analysis.
Replication	Does not apply, since bioinformatic analyses were performed. The availability of analysis scripts ensures reproducibility.
Randomization	Datasets were analyzed and integrated into the ArcPP in a random order.
Blinding	Does not apply, since bioinformatic analyses were performed.

Reporting for specific materials, systems and methods

We require information from authors about some types of materials, experimental systems and methods used in many studies. Here, indicate whether each material, system or method listed is relevant to your study. If you are not sure if a list item applies to your research, read the appropriate section before selecting a response.

Materials & experimental systems

n/a	Involved in the study
☒	☐ Antibodies
☒	☐ Eukaryotic cell lines
☒	☐ Palaeontology
☒	☐ Animals and other organisms
☒	☐ Human research participants
☒	☐ Clinical data

Methods

n/a	Involved in the study
☒	☐ ChIP-seq
☒	☐ Flow cytometry
☒	☐ MRI-based neuroimaging
